# Turning the table toward plant-based: different perspectives on menu reformulation in food service

**DOI:** 10.3389/fnut.2025.1601979

**Published:** 2025-07-24

**Authors:** Ada Rocha, Claudia Viegas, Sara Gonçalves

**Affiliations:** ^1^Green UPorto Sustainable Agrifood Production Research Centre, Faculty of Nutrition and Food Sciences, University of Porto, Porto, Portugal; ^2^Centro de Investigação em Saúde e Tecnologia, Escola Superior de Tecnologia da Saúde, Instituto Politécnico de Lisboa, Lisbon, Portugal; ^3^Faculty of Nutrition and Food Sciences, University of Porto, Porto, Portugal

**Keywords:** canteen, catering, menus, healthy food, sustainability

## Abstract

Scientific evidence supports the need to adapt food systems, counteracting the current scenario characterized by excessive and growing consumption of animal products that translates into high environmental impact, unsustainability, and negative effects on health. The food services are responsible for a high and increasing carbon footprint, emerging as a sector that promotes food sustainability through the adaptation of its operational processes and food supply. The main objective of this research was to evaluate the impact of menu reformulation and changes in the food offer on consumers’ perception and acceptance at a food service unit. A study with two stages was conducted using a convenience sample of canteen consumers (*n* = 55), and canteen employees (*n* = 6) to which a self-completion questionnaire was applied. Participants were mostly female (76.2%) and had a mean age of 40 ± 9.4 years. Menus were reformulated in stage one; stage two included food offer reformulation. Meat was considered the most important food group in both studies. Food and nutrition were highlighted as important to consumers (>80%) with statistically significant differences (*p* < 0.05). Most participants did not notice the changes made in the study (>55%). Only 32.4% of the participants were satisfied with the proposed changes, but approximately 65.8% were willing to keep the suggested changes in the future. The results of this study present clues and opportunities for strategies to change the food offer of the food service aiming to healthier and more sustainable meals.

## 1 Introduction

Food production and consumption have a major impact on the environment (1–8). Current estimations show that food production is responsible for about 25–30% of greenhouse gas emissions (8–11), 70% of (fresh) water use and 40% of land use (10, 11). Considering the prospects for population growth in the coming decades (10–12), it is expected that these rates will increase with devastating consequences for the planet (10, 11), and the increase of malnutrition, both due to lack of access to food and excessive consumption of low nutritional value food (13).

Between 1961 and 2009, the availability of animal protein sources increased by 59%, while plant-based protein sources grew only by 14% and, the demand for animal protein is expected to increase by 80% until 2050 (11). When compared to plant-based alternatives, particularly pulses, animal protein is the least sustainable food source (2–4, 10, 11), resulting in 20 times higher greenhouse gas emissions per unit of protein for consumption (8).

The last National Portuguese Food Survey showed that the dietary patterns have drifted from the recommendations (14), revealing an increasing consumption of meat and processed foods, and a decrease in non-starchy vegetables, fruit and pulses (2, 10, 11). Traditionally, animal food sources are served as the central component of the dish (11, 15), particularly meat, which plays a very important role in the diet due to its association with wealth, success, conviviality and pleasure (11). This meat centrality (15, 16), contradicts the promotion of the plant-based foods which are often disregarded in the menu descriptions.

The food services sector is responsible for providing around 67 million meals per day in Europe, reaching approximately 13.2% of this population and amounting to a total of 6 billion meals per year (17, 18). Particularly in Portugal, the annual number of meals served in this sector is approximately 300 million (17). Given the demands placed on food production in the 21st century, the food services sector stands out as a relevant actor in sustainability, and in promoting more sustainable eating habits (5, 17), by reducing the supply of less sustainable foods such as animal protein sources. This sector has an important impact on consumers’ eating habits, as they are regular suppliers for a long period of time, which supports the responsibility of this sector in public health (5, 17).

Information *nudges* are reported as a way of inducing behavioral change, and choices, enabling the information provided more appealing (4). In the food service context, *nudging* can be applied to menus (19), which represent the customers’ first contact with the service, triggering expectations and possible interest. Reformulation may concern the ordering of components (20), the type, size and color of the font used (21, 22), the use of more descriptive names (20, 23, 24) or the enhancement of sensory aspects or the cooking method (20, 24). They may also exert a stronger effect on taste perception and eating behavior than nutrition information alone (20, 24) and are associated with increased consumption of the highlighted food (25). The menus should be designed creatively, including all the menu characteristics, item layout, descriptions and labels, aiming to provide information, but driving consumer focus to the food items we aim to promote (26). Some studies reported that menus designed considering the mentioned principles are important tools that can contribute to modulating food choices (10, 20, 21, 24–27). Dayan et al. (20) observed an increased popularity of the item placed in the first (55%) and second (57%) position vs. the last and penultimate position. Wansink et al. (21) discuss different strategies to increase consumer acceptance of specific menus, such as the description of the texture, taste, smell to enhance taste expectations, or the inclusion of geographical references to the dish creating proximity and familiarity with the food origin. The use of nostalgic words can also help to trigger emotions related to tradition or family. Magnini et al. (28) changed the menu’s font style and physical weight and observed that consumers start to judge restaurants by their menus, finding that italicized font in menu descriptions has a significant influence, improving consumers’ perception of quality service.

Therefore, the main objective of this study was to evaluate the impact of menu reformulation and food offer change, favoring plant-based products, on consumer perception and acceptance.

## 2 Methods

### 2.1 Study design

An experimental study case design was conducted at one food service unit from the health and social (hospital) sector leased to a private company in Porto city, that served an average of 70 lunches daily.

The study was divided into two stages: the first stage aimed to assess the impact of menu reformulation, and the second stage aimed to evaluate the impact of food offer change. For the first stage, 2 weeks’ menus were collected and analyzed by nutritionist researchers. The analysis consisted of reading the menus, going through every dish on the menu and assessing the inclusion of vegetables in the meals, as well as their variety. Most of the menus did not provide non-starchy vegetables as part of the main dish. Following this assessment researchers improved the menus, including vegetables in dishes that only provided other food groups, considering the variety and canteen management constraints. After the menu validation, the description of the dishes was reformulated, prioritizing plant food sources, and leaving animal food sources (meat, fish or eggs) as the last component. The redesigned menus were displayed at the canteen and uploaded on the institution’s website for 2 weeks. In this first approach, no adaptation was applied to the food offer, except the addition of vegetables in the dishes that were previously identified as not including this food group.

At the second stage, the two-week menu was implemented, including vegetables in all the options presented, with a greater variety and more appealing cooking methods and presentation (e.g., sauté vegetables vs. boiled, several color vegetables). This included vegetable combinations to make them more appealing and harmonious on the plate, considering colors, and varying the offer and cooking techniques. Traditional Mediterranean Diet dishes (29), which facilitate the inclusion of vegetables and reduction of meat proportion were also introduced. These changes in food proportion consisted of a 20% reduction in meat, fish and eggs, and an increase in 30% of vegetables, while the proportion of whole grains, starchy vegetables and pulses remained unchanged, aiming to achieve a proportion of 50% non-starchy vegetables, 30% of pulses and grains and, 20% of meat, fish and eggs, in the meal. To implement these changes, food service unit staff was tutored on sustainability, Mediterranean diet and cooking skills for vegetables.

### 2.2 Population and sample

This study case was performed in a food service canteen, using a convenience sample. From the canteen’ staff all personnel (*n* = 6) were included in the study, while a total of 55 consumers participated in the study.

Data collection took place between April and May 2022. Participants were asked to sign an informed consent form, which detailed that the data collected would be used only for the study’s purposes, guaranteeing their anonymity and respecting privacy and confidentiality standards.

### 2.3 Data collection instruments

For data collection, self-completion questionnaires were used. The questionnaires were adapted from a questionnaire from a previous study conducted in Coimbra with similar objectives, namely, assessing the impact of menu description reformulation (30).

The questionnaire is divided into three parts. The first part includes five questions: two questions about the frequency of canteen use; one question about the importance (rank) attributed to food groups; two questions about the perceived relation of food with health and sustainability. The second part is related to changes of the menu, assessing the perception of the changes implemented and includes five questions: one question about the reason for the implemented changes; one question about satisfaction with implemented changes; one question to order consumed food groups; one question to rate how implemented changes impact different aspects such as health, sustainability, increased consumption of plant-based food/reduction of animal food sources and one question about willing to make permanent the implemented changes.

The last part of the questionnaire included the socio-demographic characteristics (sex, age, job, and education level).

Questions that evaluate perception, importance of food groups and satisfaction used Likert scales from 1 to 7 points.

### 2.4 Data analysis

Data was analyzed using Microsoft Excel® and SPSS Statistics 27® software. Descriptive analysis included calculating mean, standard deviation, minimum and maximum values, and simple, absolute, and relative frequencies. Normality analysis was performed using the Shapiro–Wilk test, and non-parametric tests (Wilcoxon-Mann–Whitney) were used for the comparison of means between groups.

### 2.5 Ethics

This study protocol was reviewed and approved by the Ethical Commitee of Faculdade de Ciências da Nutrição e Alimentação da Universidade do Porto (No 58/2021/CEFCNAUP/2021).

## 3 Results

### 3.1 Socio-demographic characterization

Both studies included a high percentage of participants who used the canteen daily, mostly females with an average age of 40 years old with 12 or less years of education (Table 1). Around 98% of respondents attended the canteen the day before both questionnaires were applied.

**Table 1:** Socio-demographic characterization.

Socio-demographic characteristics	Step 1 (*n* = 55)	Step 2 (*n* = 41)
Sex		
Male	23.8%	19.5%
Female	76.2%	80.5%
Age (years)	40 ± 9.4	40 ± 8.2
Level of education		
Professional courses or lower	69.8%	66.7%
Bachelor’s degree or higher	30.2%	33.3%
Canteen use frequency		
Daily	76.4%	78%
3 to 4 times a week	23.6%	22%

### 3.2 Perception of food, health and sustainability

**Figure 1:**
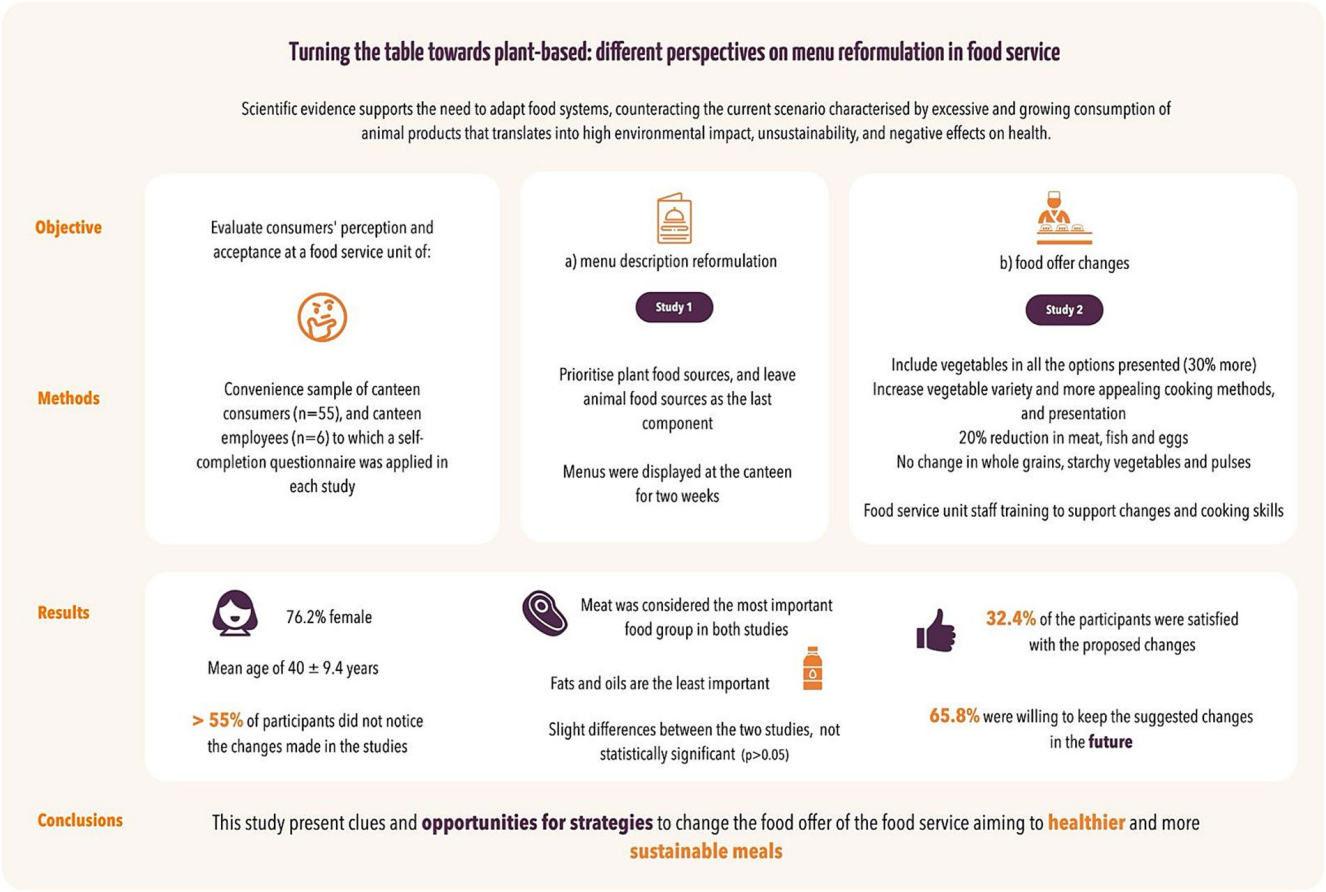
Ranking of food groups in the two phases of the study (1 = highly important; 10 = not important). S1, Study 1; S2, Study 2; WGSV, Whole grains and Starchy Vegetables; NS, non-starchy.

In both studies, meat was ranked as the most important food group, while fats were the least important (Figure 1). Slight differences between the two studies can be observed, but they were not statistically significant (*p* > 0.05).

In both stages of the study (S1 and S2), most of the participants (S1 = 89.1%; S2 = 82.9%) considered food and nutrition to be of great importance to them, but only 38.5% in S1 and 57.5% in S2 believed that individual choices in the canteen impact sustainability.

### 3.3 Changes in the menu

In the first study, only 26% of the participants noticed the changes in the menu description. From these, 45% associated them with health and nutrition and considered this to be the topic with the greatest impact on changes in the food supply.

In the second study, the majority of participants (56.4%) did not notice any changes in the food offer, namely the proportions of food served.

Those who identified the changes, associated them with health and nutrition, followed by weight control. Around 50% of individual who did not notice the changes in the first study, remained unaware int. the second study.

#### 3.3.1 Evaluation of perception and acceptance of the new food offer

The majority of participants (52.9%) evaluated perception and acceptance low (3 points or less in the scale from 1 to 7), and only 14.7% were satisfied with the new food offer. Although only 32.4% were positively satisfied with the changes, 65.8% of the participants expressed interest in maintaining them.

Most individuals (57.5%) consider that changes in the food supply contribute essentially to a more balanced diet and higher vegetable consumption.

A minority of respondents consider that the food portion change has little contribution in reducing meat consumption (35%) or fish consumption (42.5%).

### 3.4 Evaluation of perception and acceptance of the new offer—employees

#### 3.4.1 Socio demographic characterization

The questionnaire was applied to six employees, including the food unit manager. The participants have an average age of 45.2 ± 11.1 years, all female, 83.3% with 12 or fewer years of education. All participants consume their meals daily in the cafeteria.

#### 3.4.2 Changes in food supply

Employees considered sustainability to be the driver of implemented changes in food offer, and only 50% were satisfied ( $\bar{x}$ = 4.83), with no scores below 4 (1 to 7 Likert scale).

Similarly, to what was found for consumers, employees also consider the meat food group to be the most important and fat the least important. Food and nutrition are considered important by 66% of the sample.

In general, many of the employees reported that food offer changes have an impact on all the items, except for the increase in vegetable consumption. All employees considered that changes should be maintained in the future.

## 4 Discussion and conclusions

The main objective of this study was to evaluate the impact of menu reformulation and changes in the food offer, promoting plant-based products, on consumer perception and acceptance. The participants were mostly women with a bachelor’s degree or a higher education title, which was expected, considering the type of canteen (hospital unit). Most of the participants use the canteen daily, which means that they are familiar with the type of service, menus, and meals.

The ranking of food groups reveals that meat is considered the most important component in the participants’ diet, which is in line with the perception of most consumers (11, 14, 15, 31), promoted by the social norm of creating menus around animal food sources (16). Canteen employees share the same perception and, in addition to meat, fish and eggs are also highlighted. At the same time, whole-grains, vegetables, pulses, fruit, nuts and fat are considered of lower importance to the diet. While meat is socially a food with a very prestigious symbolic meaning, associated with purchasing power and physical strength, plant-based foods are seen as discreditable, linked to poverty and food shortages (16). Moreover, as a result of extensive experience preparing and cooking meat and fish, chefs and individuals are not very creative when it comes to preparing and cooking plant-based products, which are associated with longer and harder preparation times (27). Indeed, from a very young age, consumers are encouraged to consume meat and animal products as a source of protein, which is more evident in more urban areas, perhaps due to the greater consumption of meals outside the home, high exposure and quantity of pre-cooked foods that promote their consumption (32).

This emphasis (11, 15, 29) is coupled with the need to reduce meat consumption both because it is less sustainable (2–4, 33) and because of the negative impact that its high consumption has on health (13, 34–36). Protein is one of the essential nutrients in the diet and can be obtained through the consumption of various foods, but meat is the most common source, although other sources, particularly of plant origin, are considered healthier and more sustainable (37). The current (32, 37) consumption of fruit and vegetables is lower than recommended (14), emphasizing the need to change current food patterns by promoting the consumption of vegetables, pulses, nuts and seeds and whole-grains, promoting more sustainable dietary patterns such as the Mediterranean Diet (38–41) and the Planetary Health Diet (3, 42). Positive health and environmental impacts could be achieved by changing the current view, based primarily on the importance of animal food sources, particularly meat.

Fats are the food considered least important by the participants among the various food groups, something that is partly due to the link between fat and negative health effects, such as cardiovascular diseases, that have been highlighted over the years (34, 43). However, it is important to note that there are different types of fat with different effects on our bodies. The main source of fat in the Mediterranean diet is olive oil, whose composition includes monounsaturated fatty acids and antioxidants that have positive effects on health, particularly the protective effect against coronary heart disease, some types of cancer and cognitive decline due to aging (44). It is therefore a group of foods that should be given importance, but in which olive oil should be chosen as the fat of choice (44), in order to obtain the health benefits of its consumption.

In general, participants considered food and nutrition important for themselves and for their health. However, a significant percentage do not consider that their choices have an impact on sustainability. This may be due to the emotional connection that consumers have with the consumption of animal food products, mainly meat (45, 46). A study that aimed to identify barriers to reducing meat consumption found that many consumers are skeptical of the need to reduce meat consumption, value the sensory quality of meat, and do not have the cooking skills needed to prepare meat-free meals, expressing a desire to have control over their food choices (45).

Also, taste perception has the largest impact on consumers’ choices, affecting the maintenance of unbalanced eating habits (47). Therefore, the attractiveness of a dish can be guaranteed through more appealing descriptions and presenting the dish in a more agreeable way (21, 30). Nevertheless, consumers’ satisfaction with the new food offer was lower than expected. A study by Atwood et al. (10) that used the ‘decoy effect’ also failed to confirm the initial hypothesis that a price-based decoy strategy could encourage diners to shift their food choices away from meat toward plant-based alternatives. A possible explanation for the low satisfaction may be the strong predisposition for meat preference (46). Another strategy could be the incorporation of more Mediterranean dishes, which incorporate all food items in one pot, facilitating the reduction of animal protein sources inclusion and allowing for more diverse, appealing and accessible cooking techniques, promoting the consumption of plant-based products (48).

Contrary to expectations, most consumers (74%) did not perceive the change in the menu’s description, as reported in a similar study, in a public canteen in Portugal (49). This might be because regular users are used to menu cycles and the type of food regularly provided. However, those who perceived it highlighted that the adjustments carried out at this stage of the study were due to concern for “health and nutrition,” which may reveal some awareness of the importance of dietary patterns on health promotion (50). However, the changes were not related to sustainability, which may indicate that the concept of sustainability may not be clear to the population and that the food impact on sustainability may not be fully perceived by consumers, which is an interesting topic to explore in the future.

In the second phase of the study, most participants still did not notice the menu changes. However, those who identified them presented a low level of satisfaction. Once again, this result may be due to the importance given to animal food sources in the diet (11, 15, 31). The low level of customer satisfaction may also be related to changes made in the plating (vegetables served on the plate instead of as a side dish), preconceived ideas related to vegetables and the association with the reduction of meat offered to control costs. As stated before, meat is associated with the increased power of purchase (51, 52) and, while consumers may value the increased vegetable availability, either on the menu or on the plate, that they associate to health, the actual reduction of the meat portion size on the plate (that was not previously perceived on the menu description), may play a more complex role on emotions and expectations.

Most participants considered that changes in food offer contribute essentially to a more balanced diet and increase vegetable consumption (57.5 and 50%, respectively), which may be a positive point for raising awareness to the fact that the Portuguese population consumes non-starchy vegetables below the recommendations (14, 41). According to the results obtained, participants associated the change in supply with the need to promote their consumption.

Despite the majority scoring changes negatively, most participants (65.8%) expressed interest in maintaining the changes, particularly in increasing the variety of vegetables available, which might be related to the recognition of the importance of nutrition and plant-based food patterns on health (46) and may be a good indicator for future interventions in canteens. Another explanation may be the influence of social desirability, which is understood as the adoption of a culturally accepted stance by avoiding criticism when exposed to tests (53). Therefore, the acceptance of the proposed changes may have the effect of social desirability on the participants in the study, trying to get closer to the eating habits promoted.

The evaluation of the perception and acceptance of the changes of food offer was also applied to the employees of the canteen. Although they also considered meat to be the most important food group, these participants associated the changes with sustainability and half of the respondents were quite satisfied with them. It is interesting to identify this perception from this group, which is less educated, a social-demographic characteristic usually associated with low food literacy (54). To implement the changes in the food offered, this group was tutored on sustainability, Mediterranean diet and more appealing ways of cooking vegetables. Although the importance of meat was highly scored, probably due to emotional reasons already explained, this group might have had a good understanding of the sustainability issues related to meat and vegetables. Therefore, this might be an important stakeholder to consider for permanent changes in the food service offer.

### 4.1 Limitations and strengths

Some of the respondents associated the questionnaire with the service provided by the company and not with the purpose of the study, which might have had an impact on the responses, namely the ones related to satisfaction. This study used a convenience sample, impairing the generalization of the results. Nevertheless, convenience samples are widely used in exploratory research because they are cost-effective and easy to obtain (55). Also, the consumers of this cafeteria are similar to some food service adult consumers (56). Other food contexts, such as industry or technology, include mostly men and, therefore, these results may not apply as much.

To our knowledge, this is one of the first studies to act on the food offer change, aiming to reduce meat offer and increase plant-based food sources, aiming to understand its viability and impact on sustainability. It provides insights into consumers’ and food service staff’s perceptions and acceptance for future studies.

## 5 Conclusion

This research was intended to promote new practices to change perceptions and encourage sustainable consumption. Changes in menu descriptions were not perceived by consumers, and in general, changes in the food offered were accepted. Consumers and employees showed interest in maintaining the proposed practices, which constitutes a window of opportunity for future interventions in food service toward sustainability.

## Data Availability

The raw data supporting the conclusions of this article will be made available by the authors, without undue reservation.
